# Supplementary material for: Hierarchical assembly of tryptophan zipper peptides into stress-relaxing bioactive hydrogels
Source: Nat Commun. 2023 Oct 23;14:6604. doi: 10.1038/s41467-023-41907-1 (PMC10593748; doi:10.1038/s41467-023-41907-1)
Supplement: Supplementary file 1 — Supplementary Information [file 41467_2023_41907_MOESM1_ESM.pdf]

Supplementary Information

Hierarchical assembly of tryptophan zipper peptides into self-healing bioactive hydrogels

Ashley K. Nguyen[1,2], Thomas G. Molley[1,2,3], Egi Kardia[4,5,6], Sylvia Ganda[1,2], Sudip Chakraborty[1], Sharon L. Wong[4,5,6], Juanfang Ruan[7], Bethany E. Yee[1,2], Jitendra Mata[8], Abhishek Vijayan[3,4], Naresh Kumar[1], Richard D. Tilley[1,7], Shafagh A. Waters[2,4,5,6,9], Kristopher A. Kilian[1,2,3,6*]

Correspondence to: k.kilian@unsw.edu.au

This PDF file includes:

Supplementary Figures 1–23
Supplementary Table 1

Other Supplementary Materials for this manuscript include the following:

Supplementary Movies 1–5

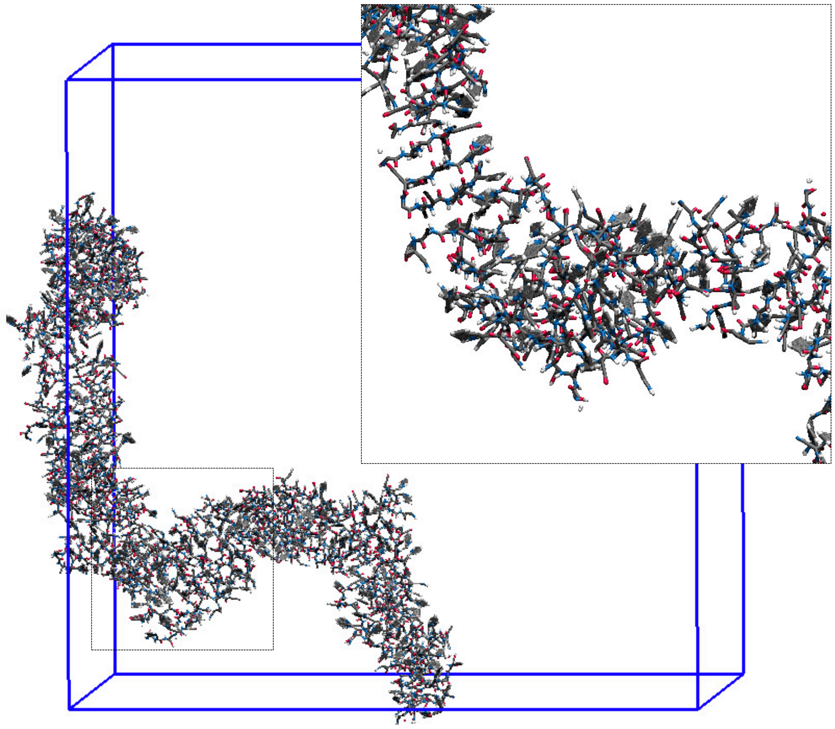

Supplementary Figure 1. Trpzip-V nanofiber backmapped from coarse-grain to all-atom resolution. Inset shows close-up of Trpzip-V monomers organization within the nanofiber.

Trpzip-QV

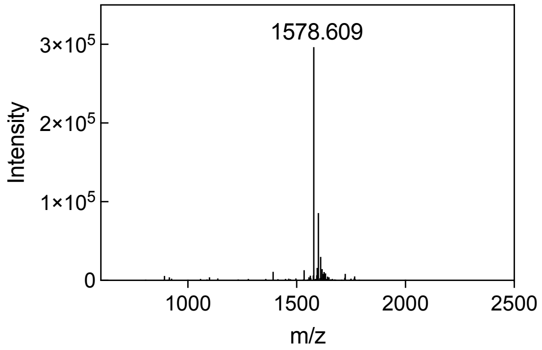

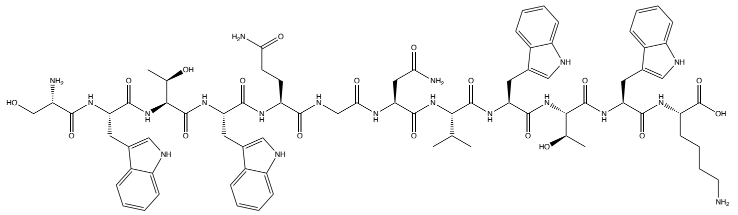

Chemical formula: $C_{77}H_{99}N_{19}O_{18}$
Exact mass: 1577.74

Trpzip1

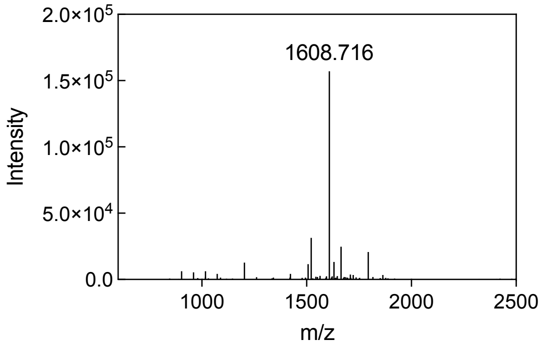

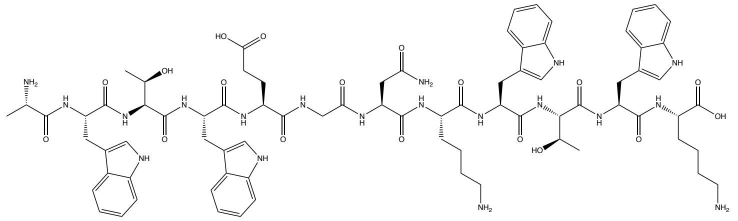

Chemical formula: $C_{78}H_{101}N_{19}O_{19}$
Exact mass: 1607.75

Trpzip-QV–IKVAV

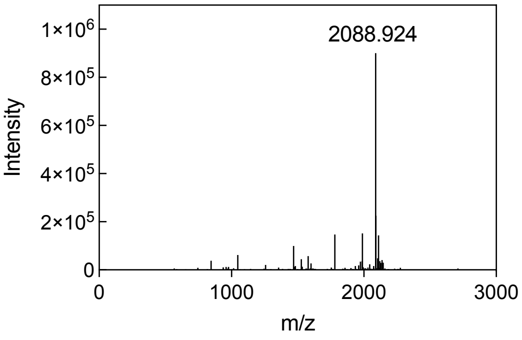

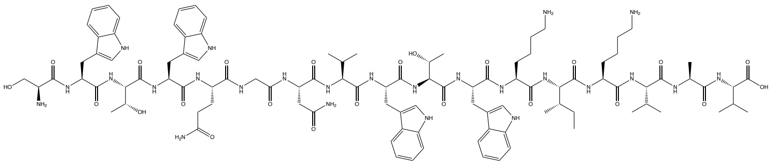

Chemical formula: $C_{102}H_{1045}N_{125}O_{23}$
Exact mass: 2088.09

Supplementary Figure 2. MALDI-TOF mass spectra (left) and corresponding chemical structure, chemical formula, and exact mass (right) of Trpzip peptides synthesized.

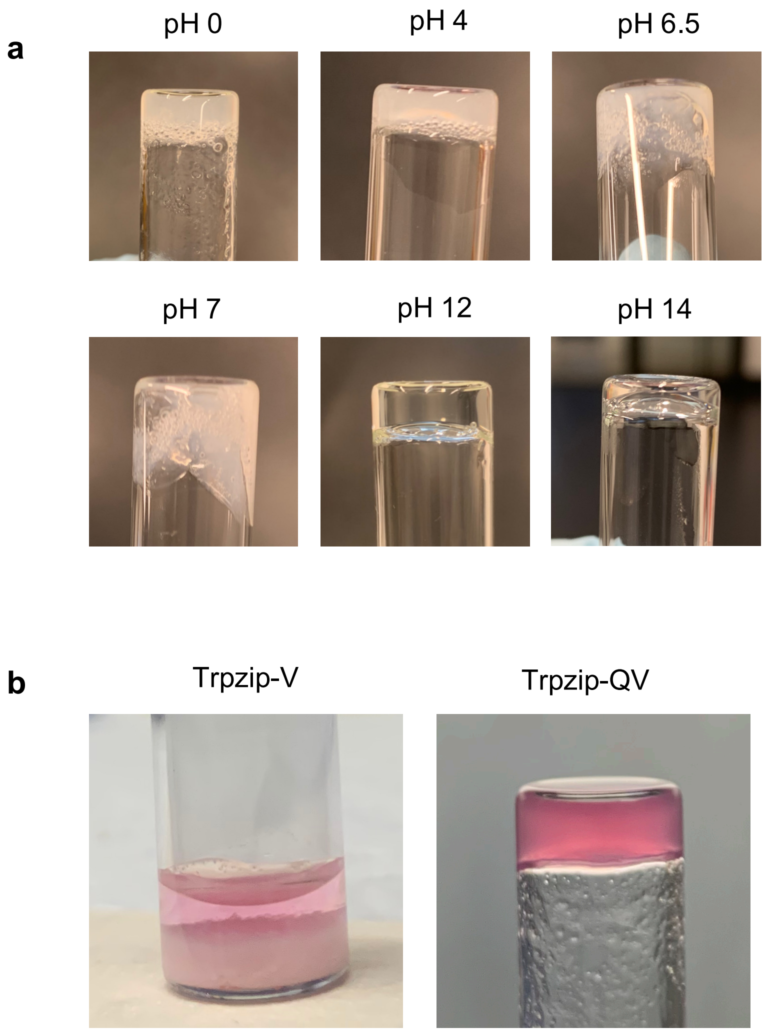

Supplementary Figure 3. The effect of pH on the gelation of Trpzip variants. (a) Hydrogel formation of Trpzip-V across a range of pH conditions. **(b)** The gelation of Trpzip-V (net charge 0 at pH 7) at pH 7 in DMEM compared to Trpzip-QV (net +1 charge at pH 7).

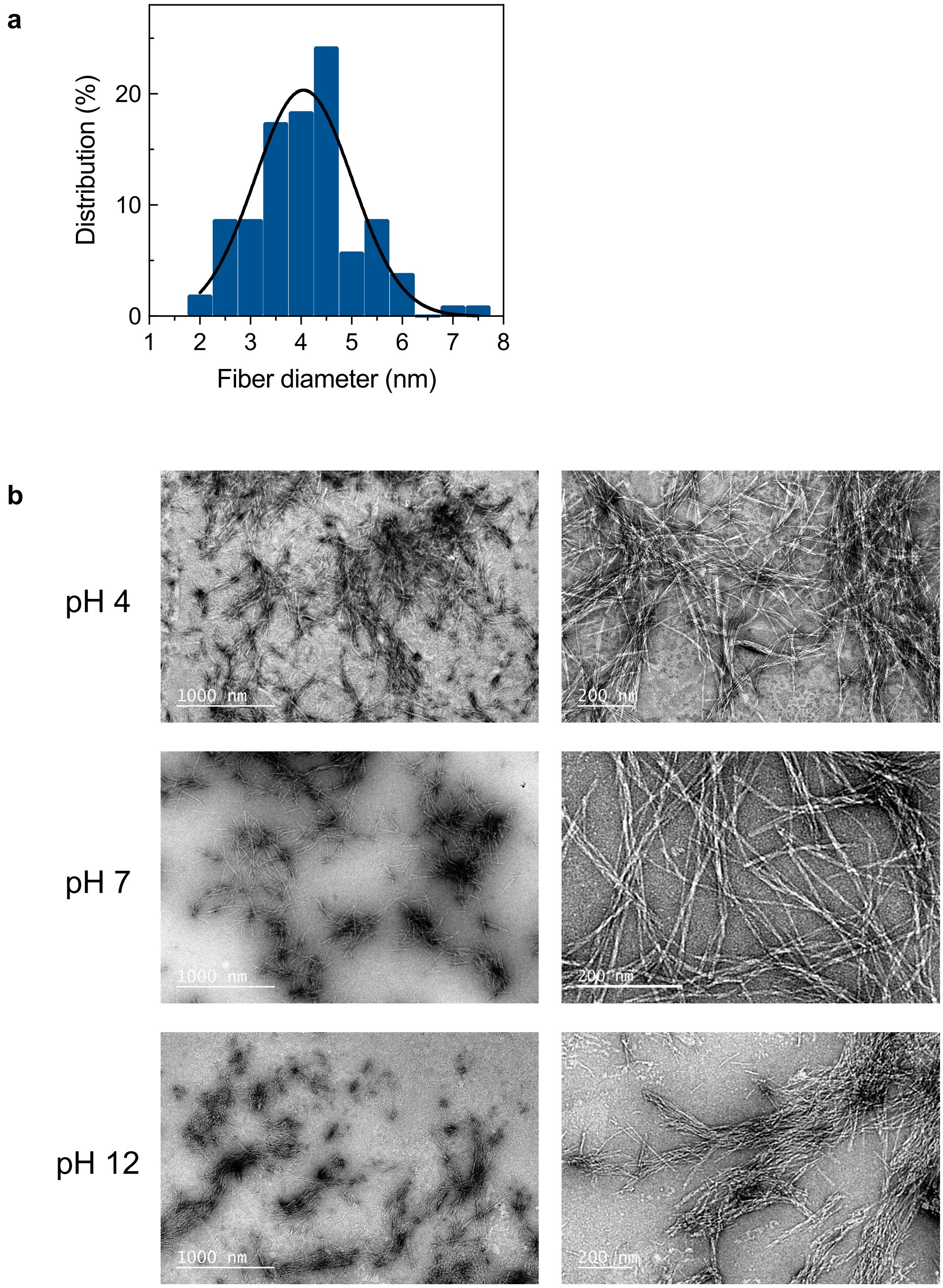

Supplementary Figure 4. Nanofiber formation of Trpzip-QV peptides. (a) Frequency distribution of average fiber diameter for Trpzip-QV nanofibers at pH 7. (**b**) TEM images of Trpzip-QV nanofibers formed across a range of pH values. Data is representative of 2 independent experiments.

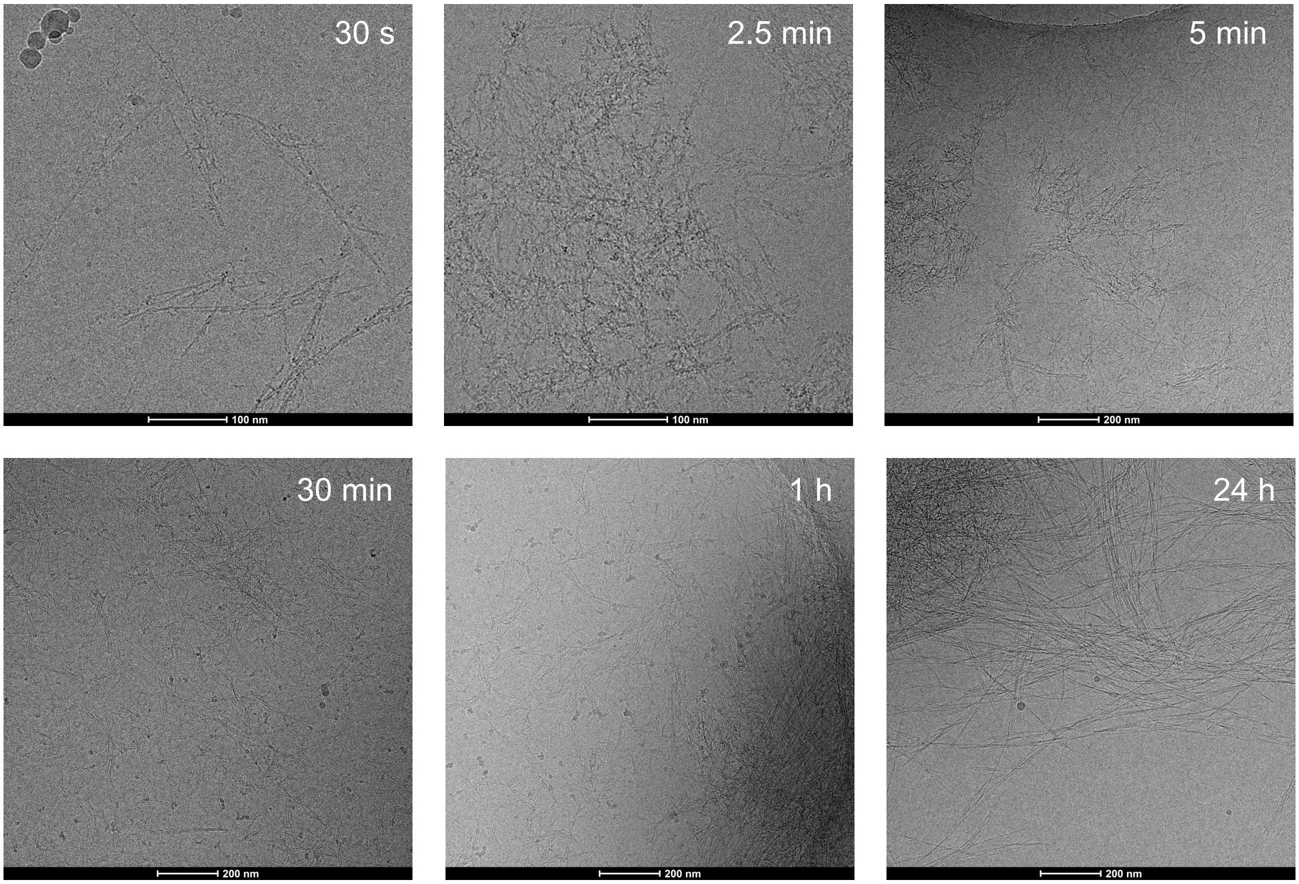

Supplementary Figure 5. Cryo-TEM of Trpzip-QV hydrogels (2% w/v) imaged at various timepoints post-gelation in DMEM, pH 7. Data is representative of 2 independent experiments.

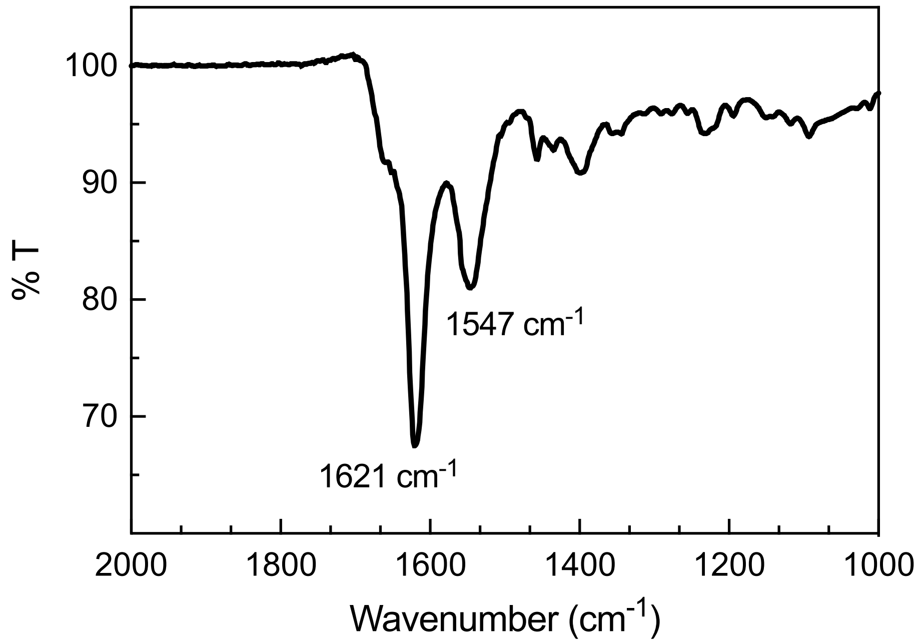

Supplementary Figure 6. FTIR spectra of Trpzip-QV hydrogel (3% w/v) prepared in DMEM, pH 7.

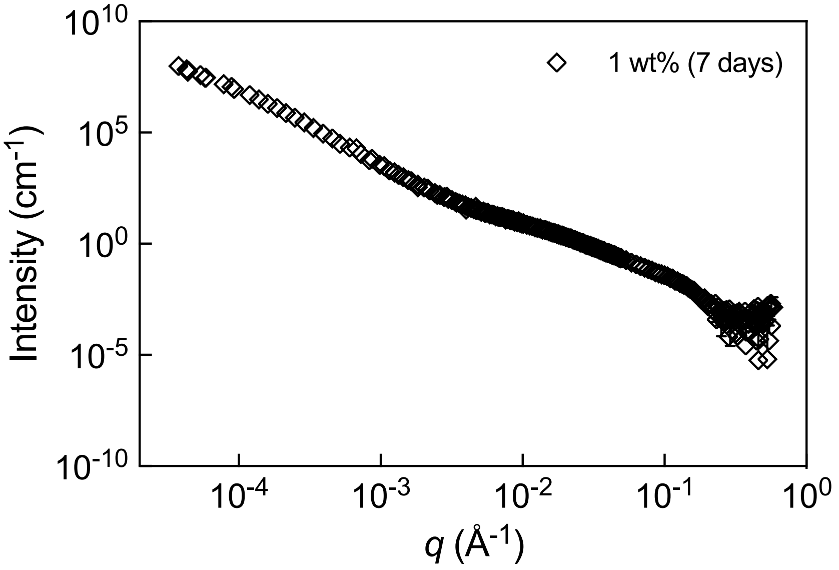

Supplementary Figure 7. USANS profile of Trpzip-QV (1% w/v) in deuterated DMEM measured after 7 days at 37 ºC.

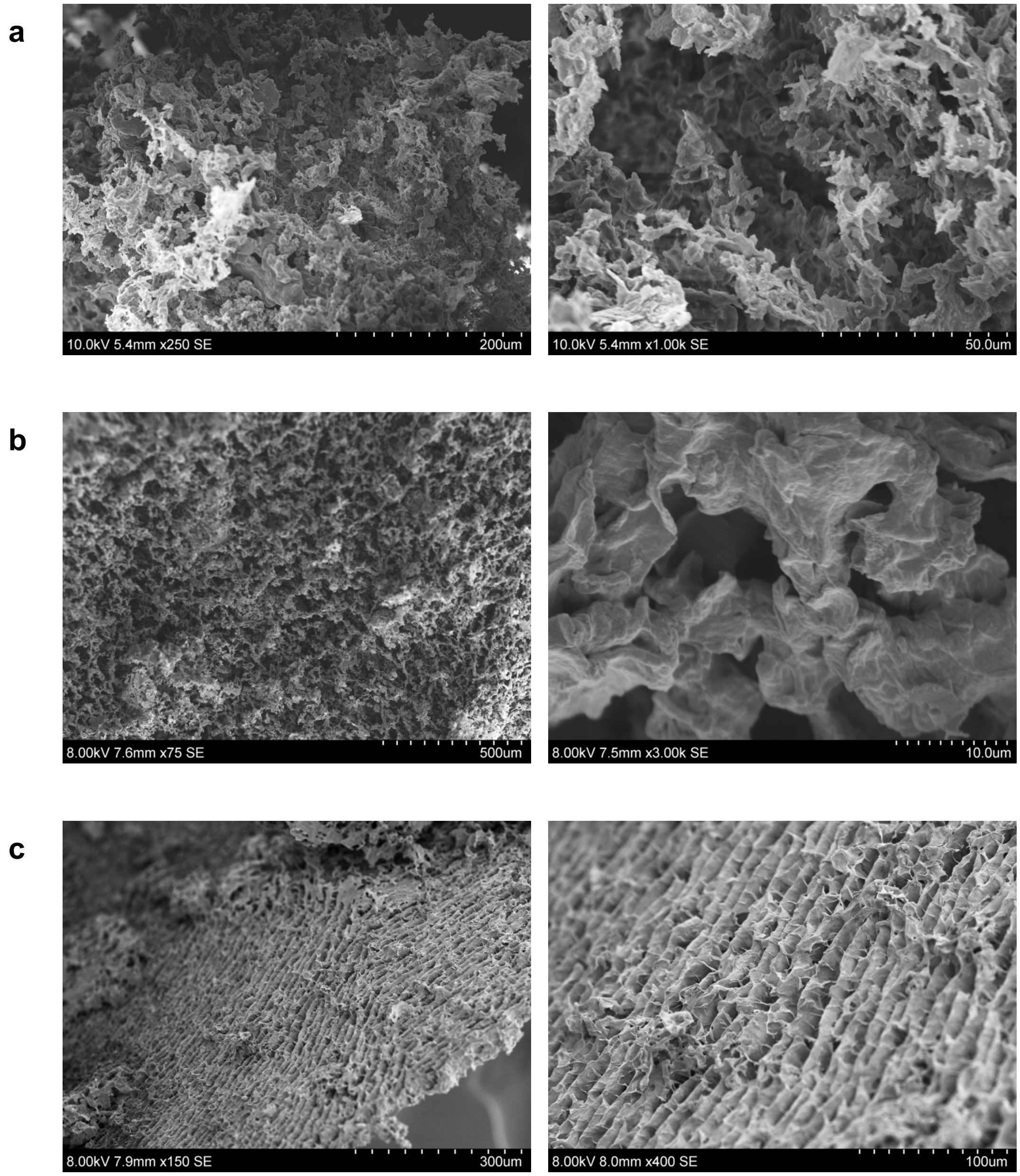

Supplementary Figure 8. Scanning electron micrographs (SEM) of Trpzip-QV hydrogels. Data is representative of 2 independent experiments. (**a**) SEM of Trpzip-QV hydrogels at 1% (w/v) at pH 7. (**b**) SEM of Trpzip-QV hydrogels at 3% (w/v) at pH 7. (**c**) SEM of Trpzip-QV hydrogels at 3% (w/v) at pH 14.

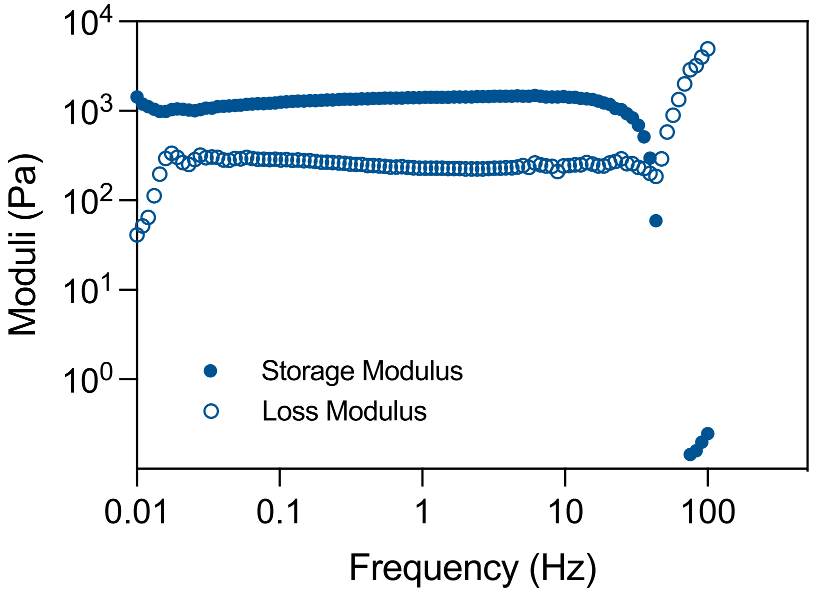

Supplementary Figure 9. Frequency sweep of Trpzip-QV hydrogel (1% w/v).

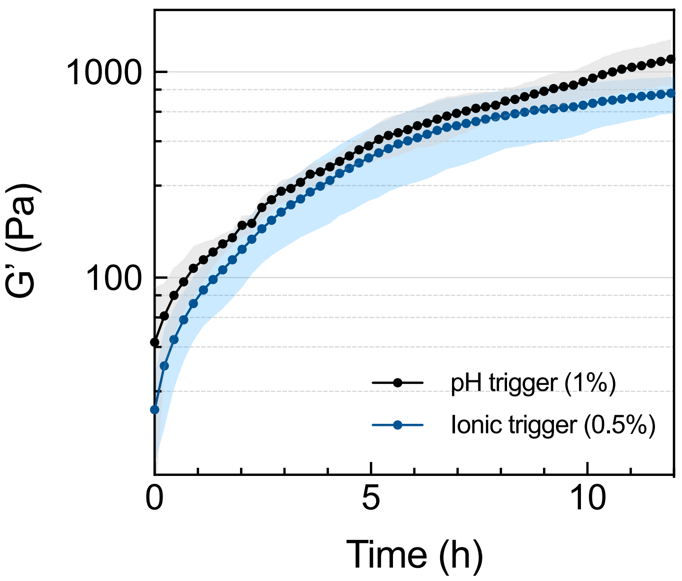

Supplementary Figure 10. Time sweeps of Trpzip gels prepared using the pH trigger method (black curve) at 1% w/v and the ionic trigger (blue curve) at 0.5% w/v. Data is represented as mean±s.d. (shaded area) from *n* = 3 independently prepared hydrogels.

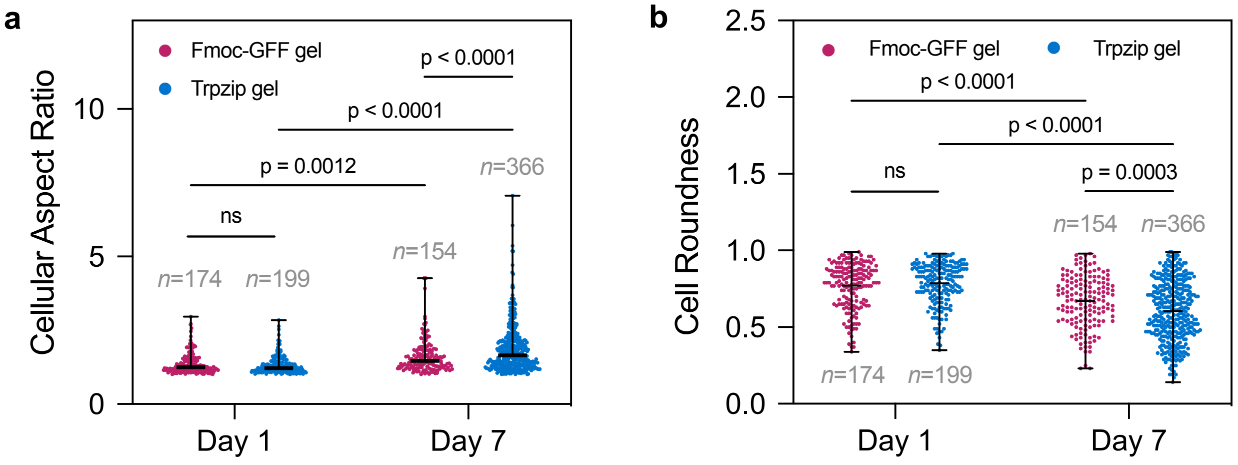

Supplementary Figure 11. Morphometric analysis of HFF cells grown in Trpzip gels (no RGD) and Fmoc-GFF gels (no RGD) after 1 day and 7 days. (**a**) Changes in cellular aspect ratio over 7 days is presented as mean±s.d. P-values were calculated using a two-way ANOVA. (**b**) Change in cell roundness over 7 days is presented as mean±s.d. P-values were calculated using a two-way ANOVA.

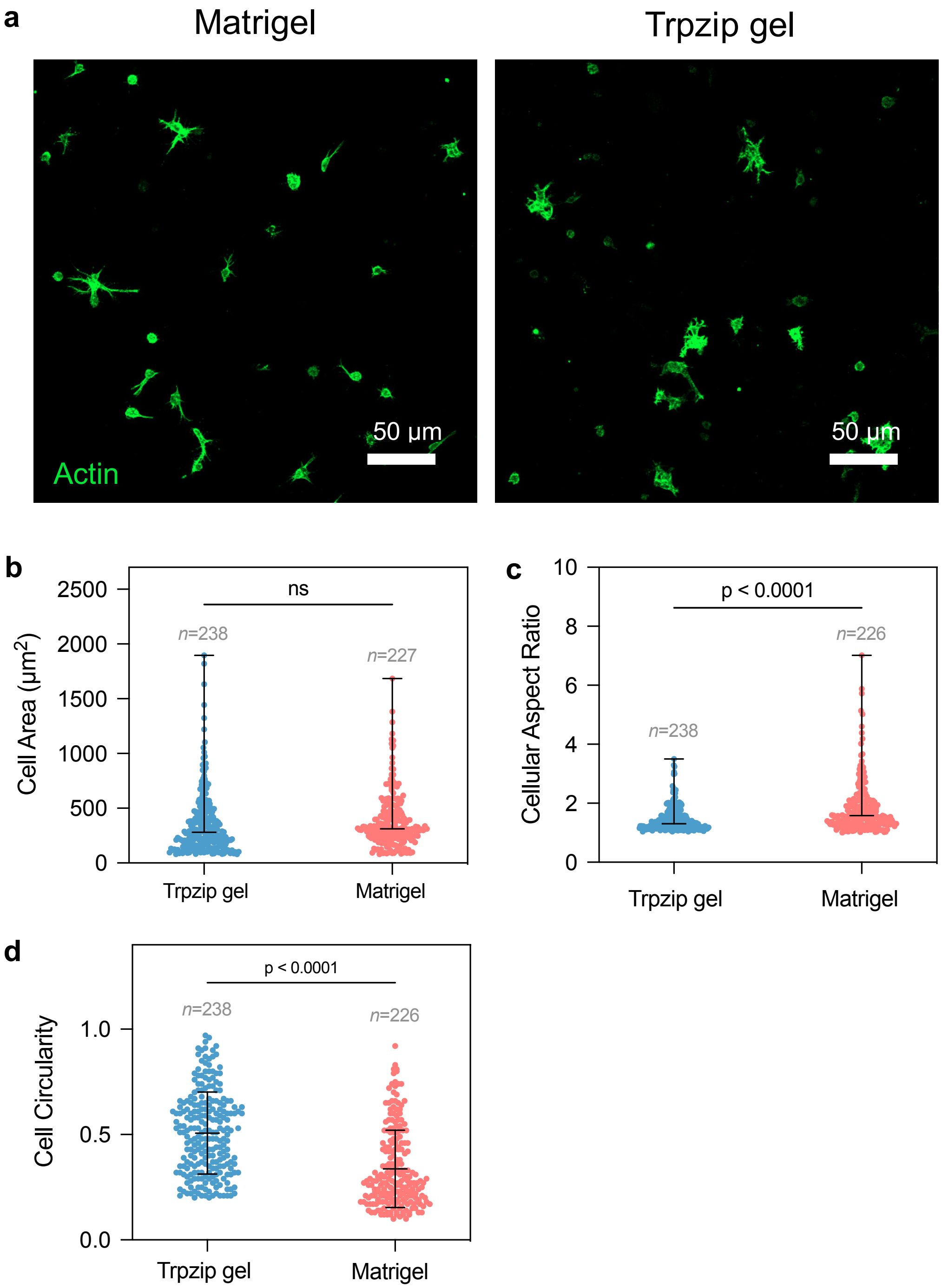

Supplementary Figure 12. Morphometric analysis of HFFs grown in Trpzip gel (no RGD) and Matrigel after 7 days. (**a**) Confocal microscopy images of HFF cells cultured in Matrigel and Trpzip gels (no RGD) for 7 days. (**b**) Quantification of area of cells grown in Trpzip gel and Matrigel. Data is presented as mean±s.d. P-values were calculated using a two-tailed unpaired *t*-test. (**c**) Quantification of aspect ratio of cells grown in Trpzip gel and Matrigel. Data is presented as mean±s.d. P-values were calculated using a two-tailed unpaired *t*-test. (**d**) Quantification of circularity of cells grown in Trpzip gel and Matrigel. Data is presented as mean±s.d. P-values were calculated using a two-tailed unpaired *t*-test.

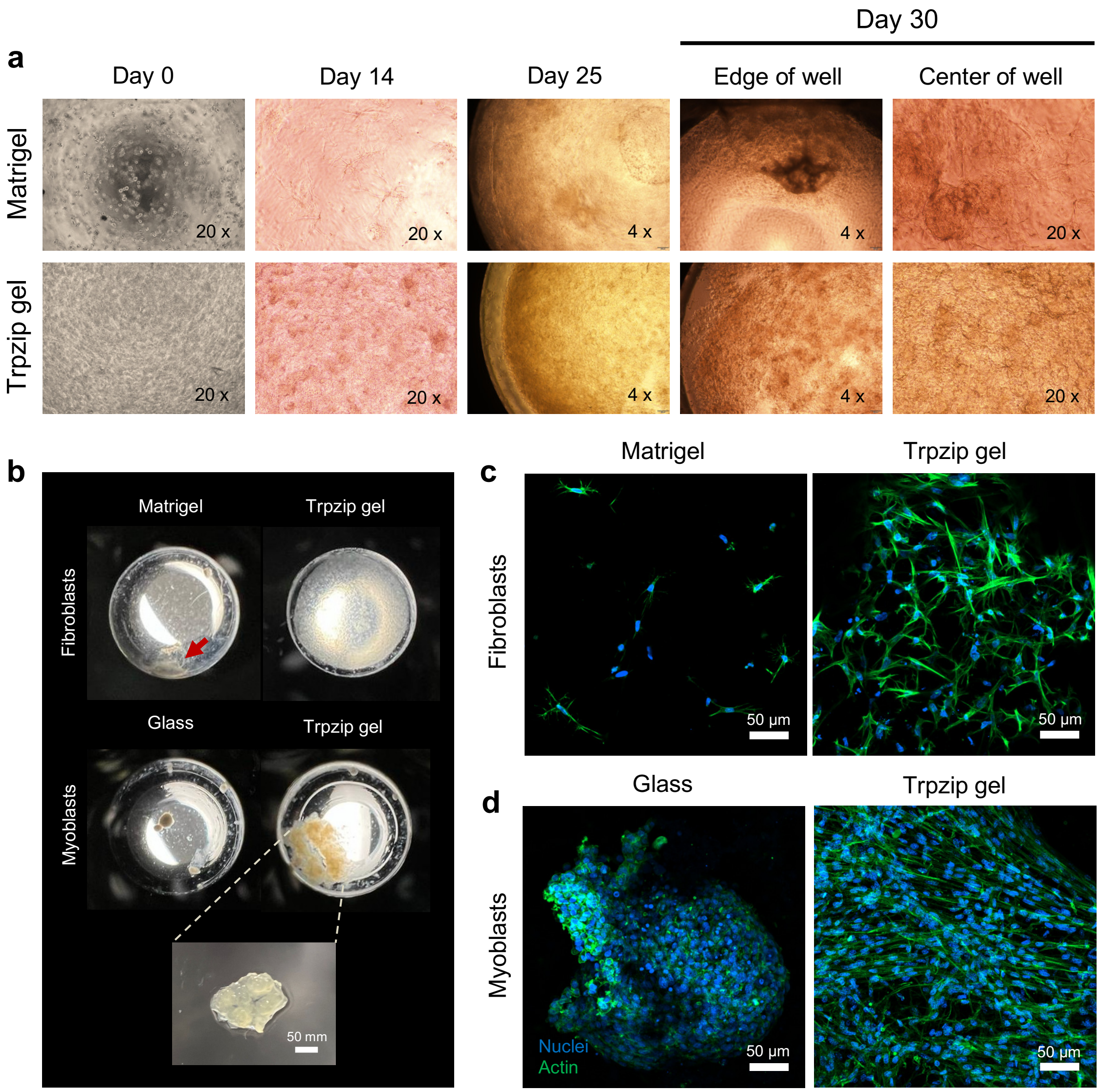

Supplementary Figure 13. Culture of mammalian cells in Trpzip gels for one month. (a) Brightfield images of fibroblast (HFF-1s) cells cultured in Matrigel and Trpzip gels, showing state of respective gels over 30 days. (**b**) Photographs of 96-well culture dish containing fibroblast and myoblast cells cultures, showing hydrogel state after 30 days. Red arrow in Matrigel sample shows section of Matrigel that has detached from glass by Day 30. Bottom photograph shows cell-laden Trpzip gel after 30 days. (**c**) Immunofluorescence images of fibroblasts cultured in 3D in Matrigel and Trpzip gels, stained for actin (green) and nuclei (blue). (**d**) Immunofluorescence images of myoblasts cultured in 2D on glass, and 3D in Trpzip gels, stained for actin (green) and nuclei (blue).

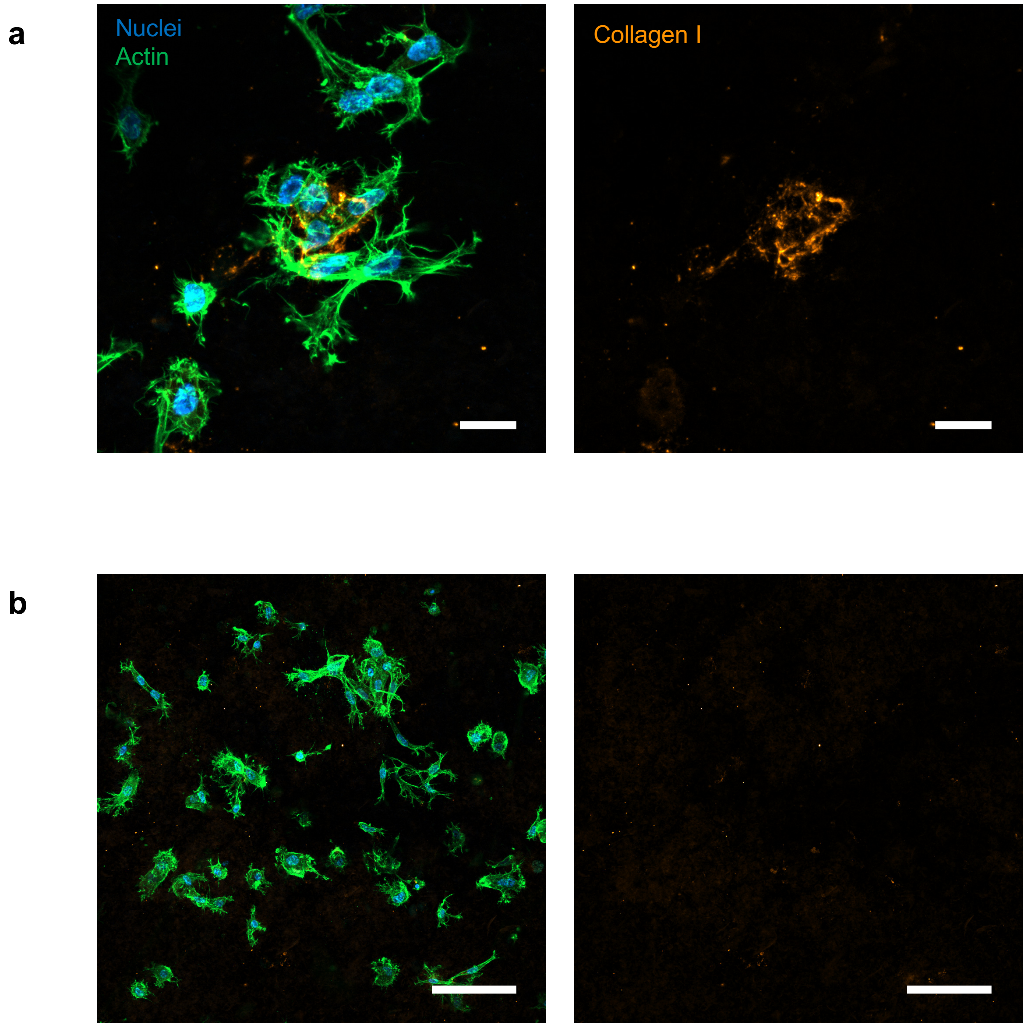

Supplementary Figure 14. Immunofluorescence confocal images of HFFs cultured in Trpzip gels (no RGD) for 14 days. (**a**) Cells in Trpzip gel immunostained with primary antibody Collagen 1 and secondary 555 antibody. Scale is 20 µm. (**b**) Cells in Trpzip gel immunostained with only secondary 555 antibody as control. Scale is 100 µm.

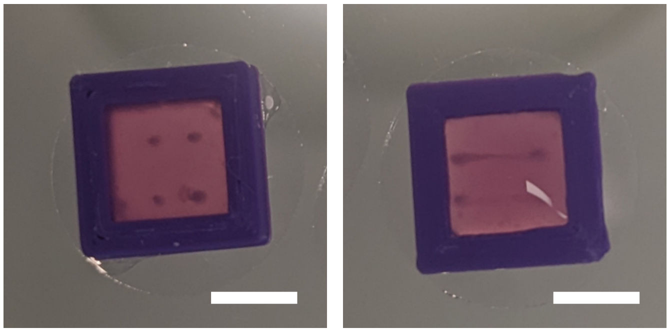

Supplementary Figure 15. Bioprinting of live cell bioink into Trpzip gel support material housed in custom 3D-printed square molds. Photograph of four droplets of cells printed in Trpzip gel (left) and two lines of cells printed in Trpzip gel (right). Scale is 500 µm.

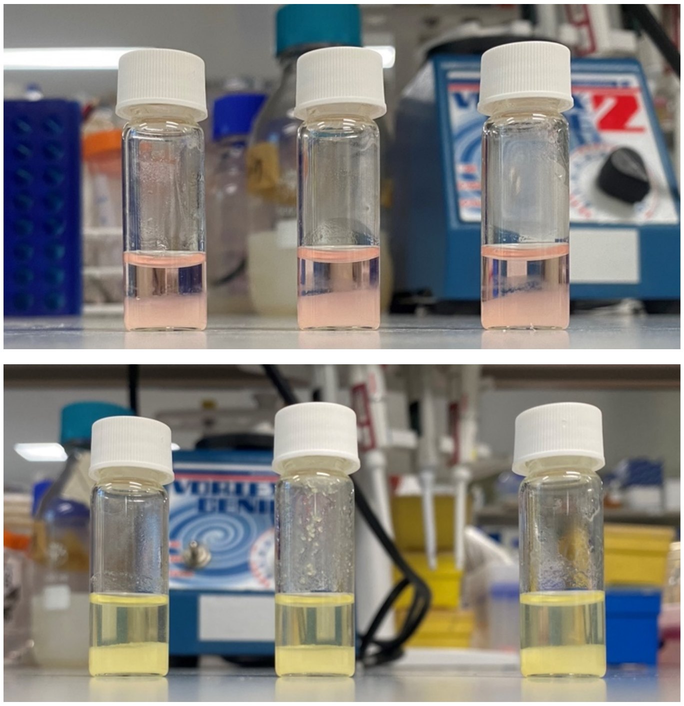

Supplementary Figure 16. Photographs of bacterial growth in the presence of antimicrobial Trpzip gels (top) and non-antimicrobial control gels (bottom) after 24 h at 37 ºC. Data is representative of 2 independent experiments.

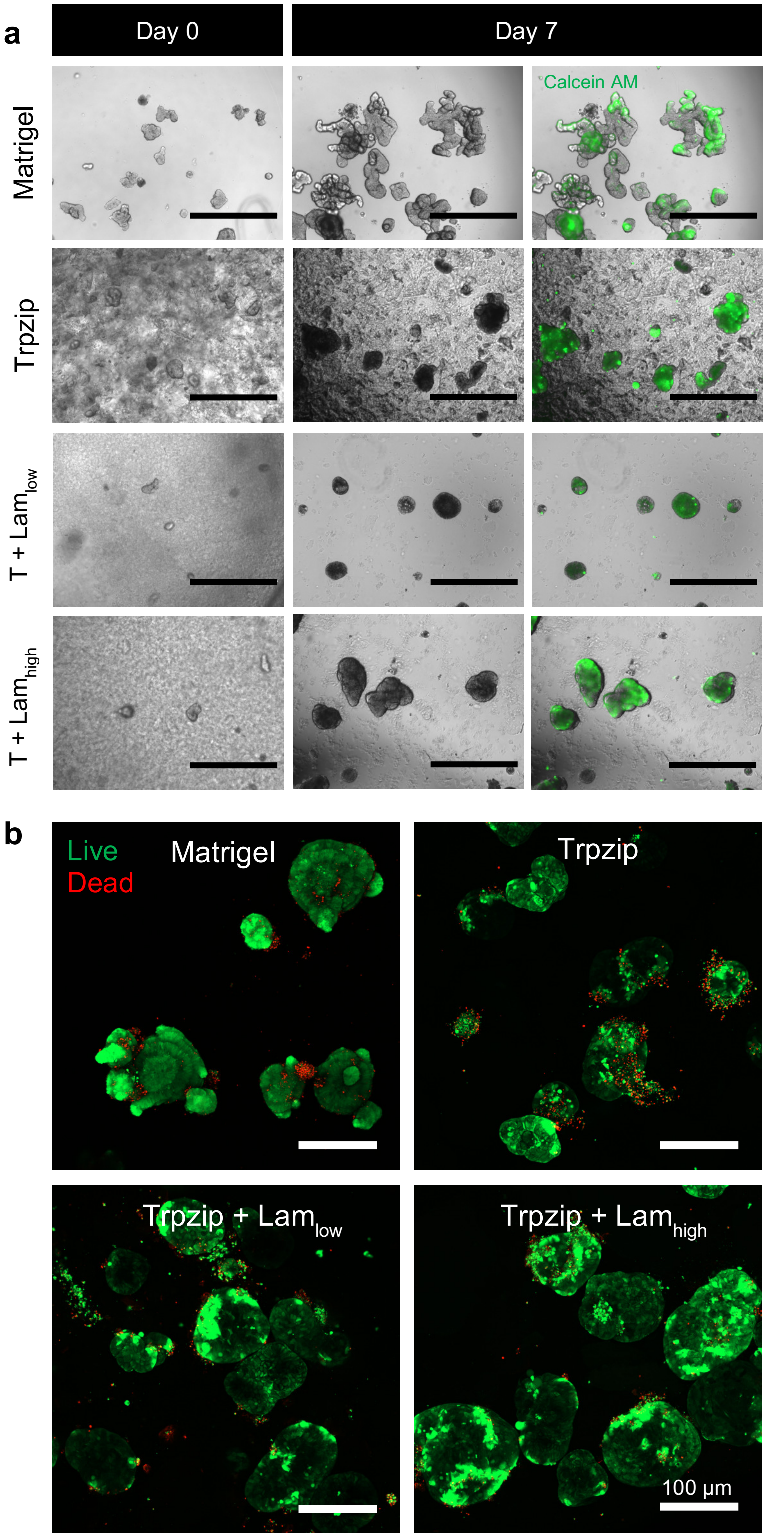

Supplementary Figure 17. Adult stem cell derived organoid growth in Trpzip gels in the presence of varying amounts of laminin. (**a**) Representative brightfield and Calcein AM imaging of organoids grown over 7 days in Matrigel, Trpzip gels, and Trpzip gels with low and high laminin content, respectively. Scale is 1 mm. (**b**) Live/dead stain of organoids cultured for seven days in Matrigel, Trpzip gels, and laminin supplemented Trpzip gels using Calcein AM (green) and Ethidium Homodimer (red).

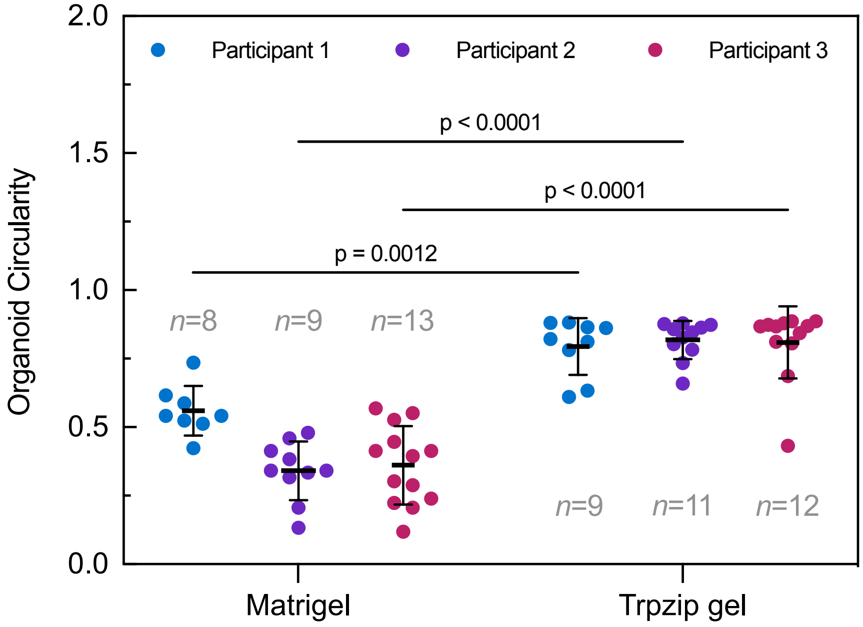

Supplementary Figure 18. Circularity of organoids following 7 days of culture in either Matrigel or Trpzip gels across three participant lines. Data is presented as mean±s.d. P-values were calculated using two-way ANOVA.

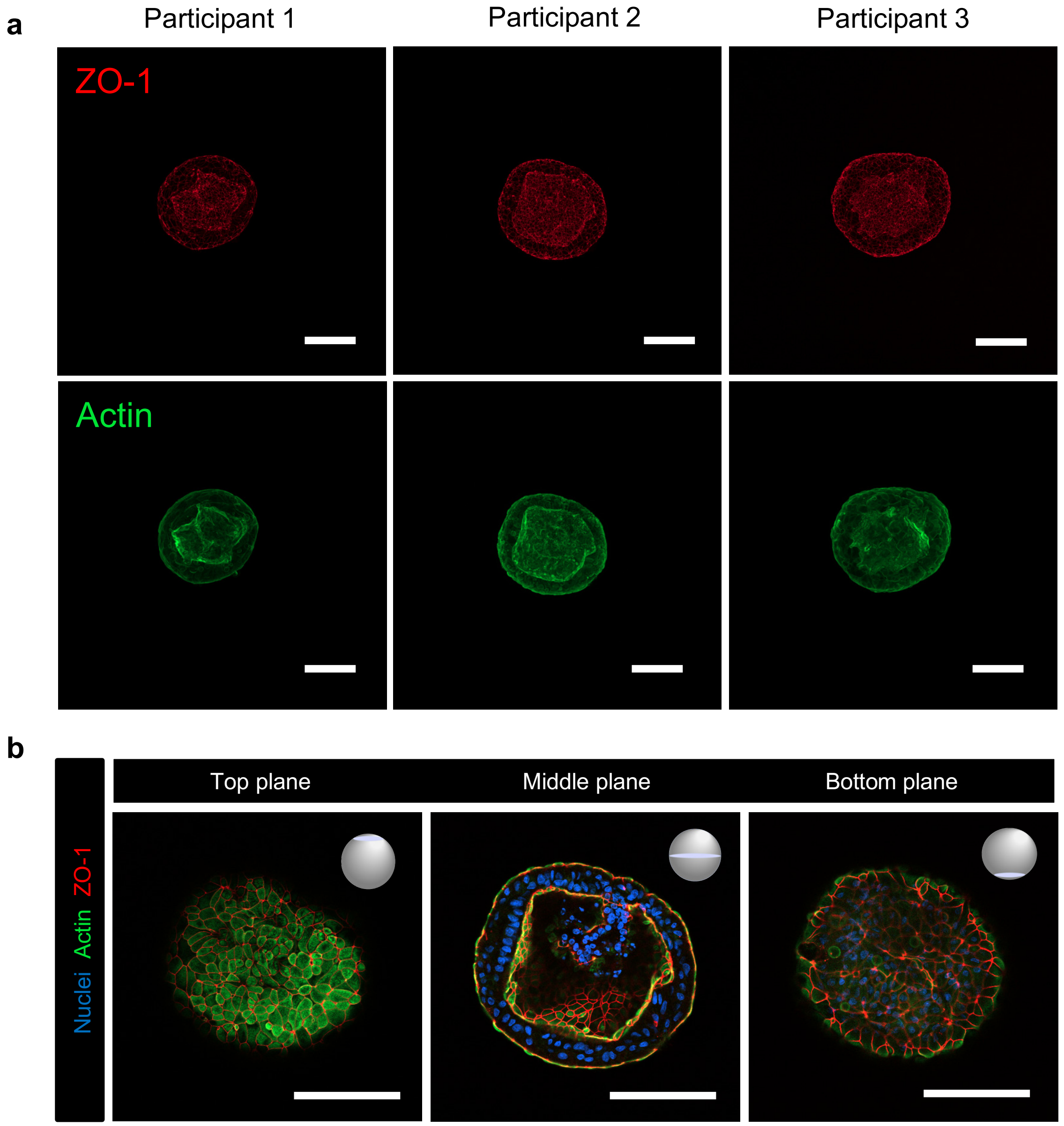

Supplementary Figure 19. Immunofluorescent confocal images of intestinal organoids grown in Trpzip gels for 7 days. (**a**) Representative max intensity projection images of organoids from three different participant lines. Scale is 100 µm. (**b**) Immunofluorescence images of one organoid taken at different z-plane heights. Scale is 100 µm.

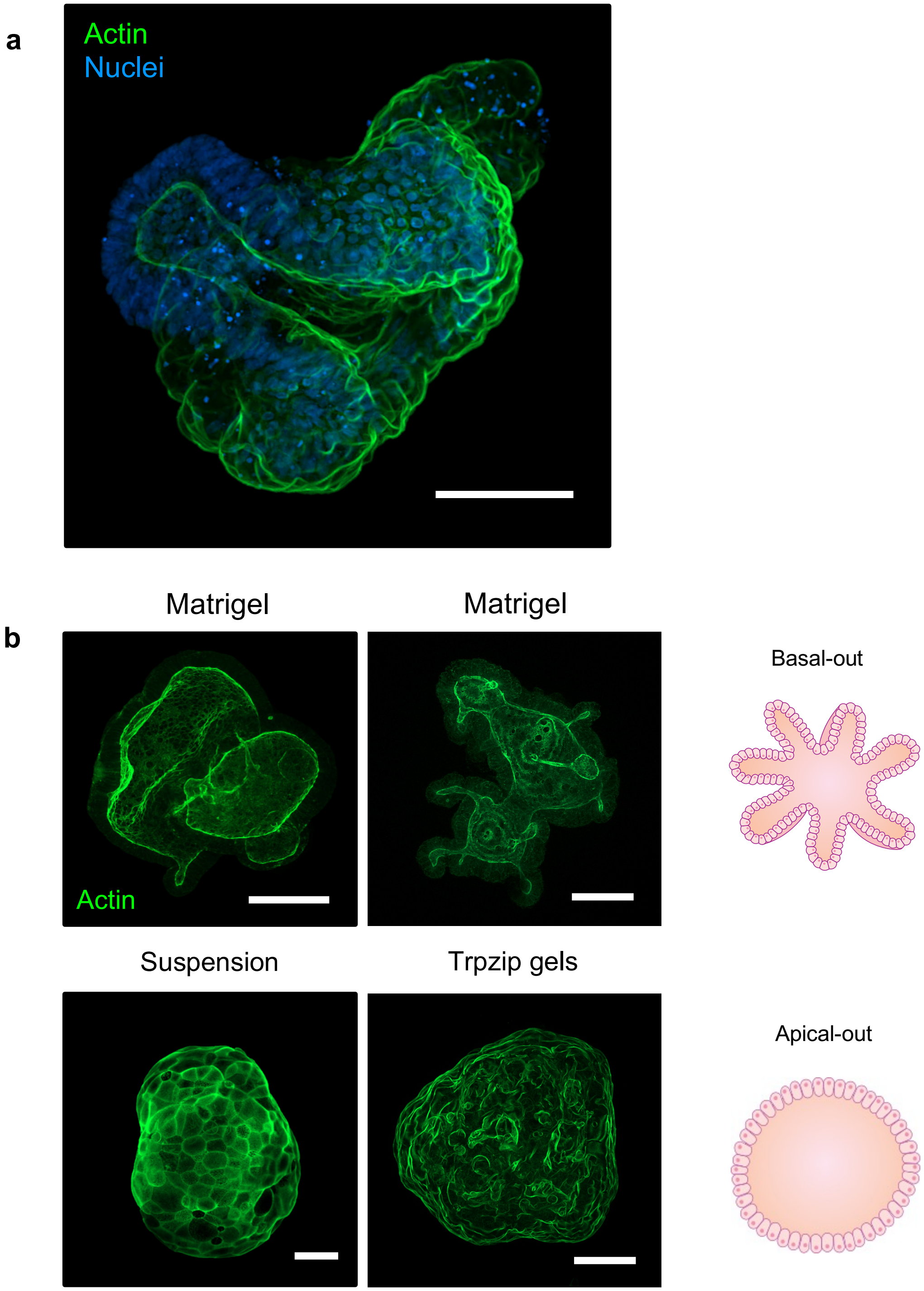

Supplementary Figure 20. Immunofluorescence confocal images of iPSC-derived intestinal organoids grown in Trpzip gel. (a) Intestinal organoid grown in Trpzip gel for 3 days showing mixed basal and apical polarity. Scale is 200 µm. (**b**) Filamentous actin structure of organoids grown in Matrigel with basolateral polarity (top left and right) compared to organoids with apical-out polarity generated from culture in suspension (left) and Trpzip gels (right). Scale is 100 µm.

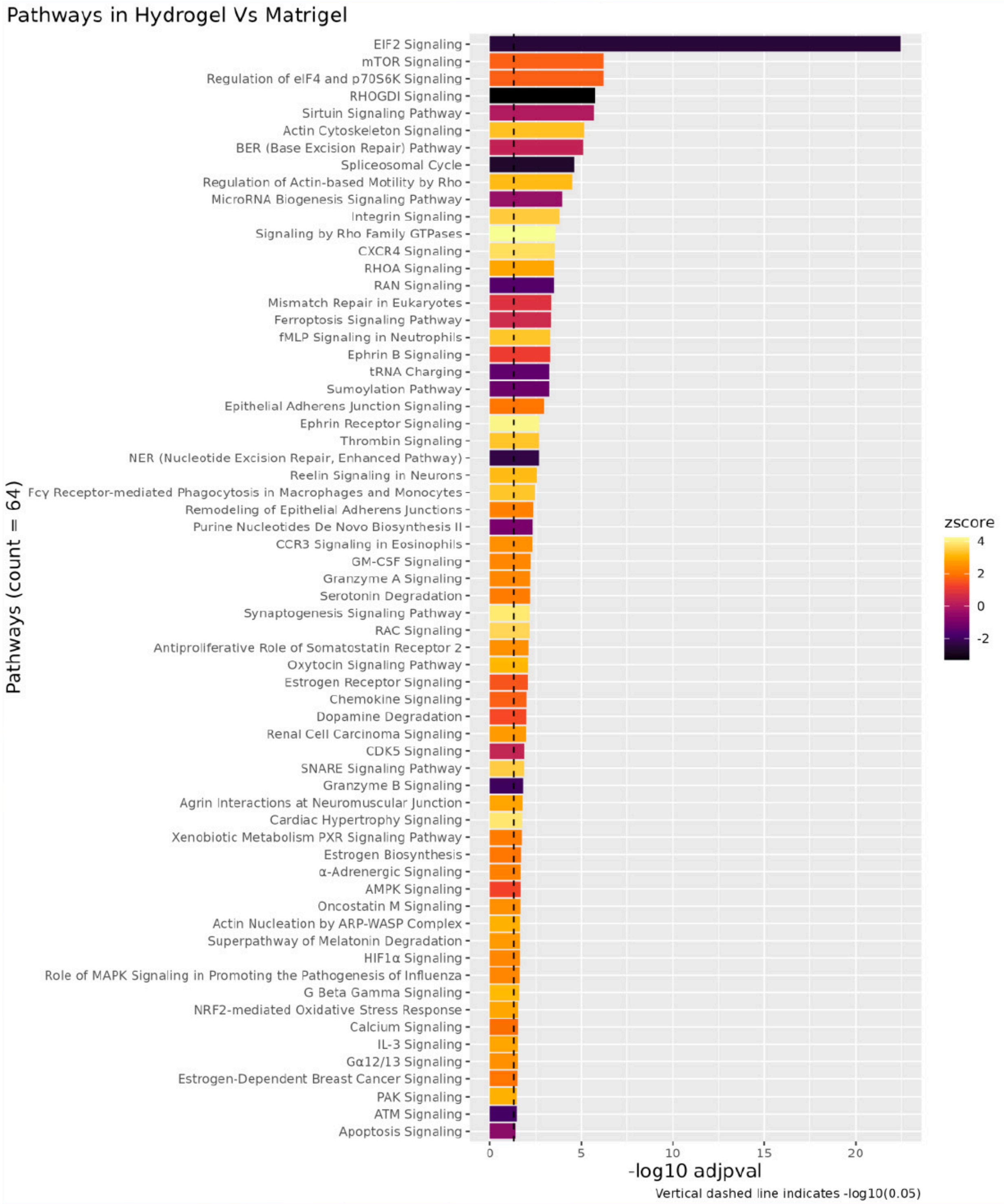

Supplementary Figure 21. Full ingenuity pathway analysis of all pathways compiled from global proteomics study.

a

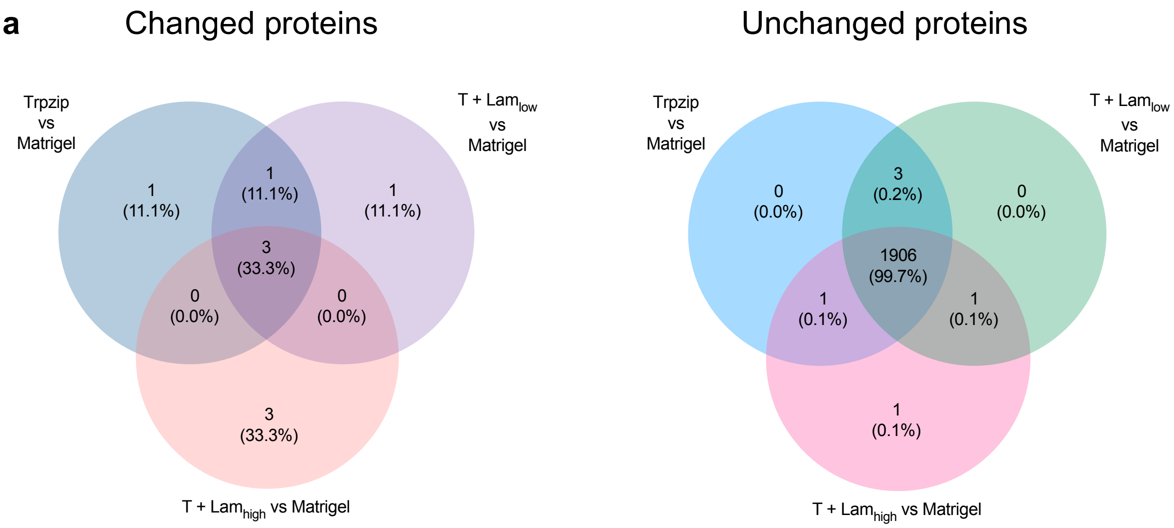

b

Euclidean distances	**Matrigel**	**Trpzip gel (T)**	**T + Lam$_{Low}$**
**Trpzip gel (T)**	120.2180		
**T + Lam$_{Low}$**	125.72881	86.83703	
**T + Lam$_{High}$**	99.00486	115.47928	121.72854

Supplementary Figure 22. Differential protein expression profiles of organoids grown in Matrigel, pure Trpzip gels, and Trpzip gels supplemented with low and high laminin content. (**a**) Venn diagrams depicting detected proteins that possessed a significantly greater expression in Trpzip gels, with and without high/low laminin and Matrigel (left), as well as those unchanged (right) between experimental groups. (**b**) Table containing Euclidean distances calculated based on hierarchical clustering analysis of global proteomics.

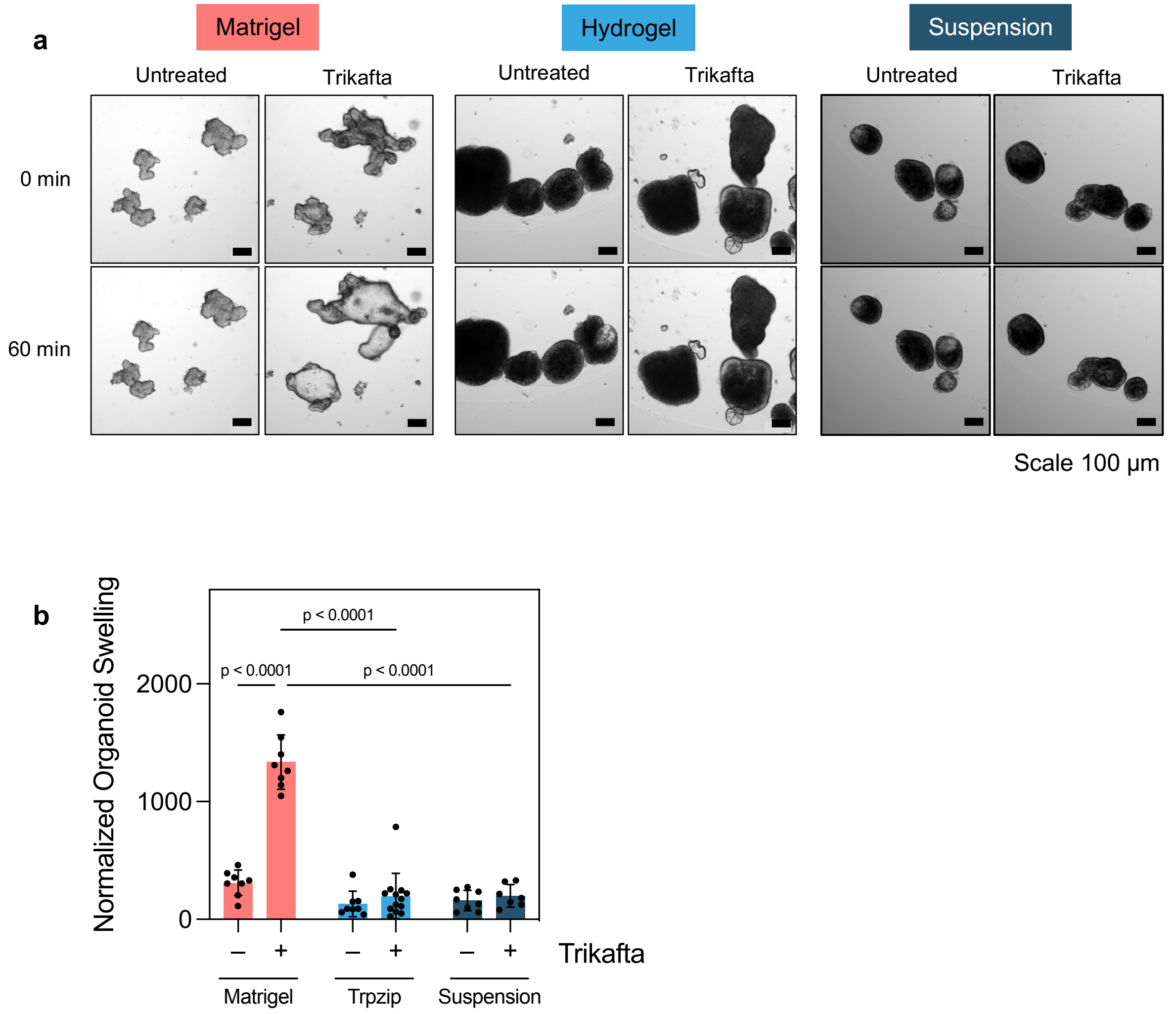

Supplementary Figure 23. Forskolin swelling assay performed on adult stem derived organoids grown in Matrigel, Trpzip hydrogels, or in suspension for 7 days. (**a**) Live brightfield imaging of organoids pre-exposure and post-exposure to CFTR activation (Trikafta treatment). (**b**) Quantification of the change in organoid size pre-exposure and post-exposure to CFTR activation (Trikafta treatment). Data is presented as mean±s.d. with a minimum of n = 8 organoids per condition. P-values were calculated using two-way ANOVA.

Supplementary Table 1. Primary and secondary antibodies used for immunostaining.

Stain	Supplier	Catalog No.	Dilution
Hoechst 33342	Life Technologies Australia Pty ltd	687117	1:500
Phalloidin-Atto 488	Sigma-Aldrich	49409	1:500
CDX2	Abcam	ab76541	1:300
MUC2	Invitrogen	MA5-12345	1:300
Lysozyme	Abcam	Ab108508	1:300
ZO-1	Abcam	ab216880	1:300
Collagen I	Abcam	ab34710	1:300
Anti-Rabbit IgG (H+L), CF™ 647 antibody produced in goat	Sigma-Aldrich	SAB4600184	1:500
Anti-Mouse IgG1 (γ1), CF™555 antibody produced in goat	Sigma-Aldrich	SAB4600302	1:500
Calcein AM	Life Technologies Australia Pty ltd	L3224	2 μM
Ethidium Homodimer-1	Life Technologies Australia Pty ltd	L3224	4 μM
