# Supplementary material for: Hierarchical assembly of tryptophan zipper peptides into stress-relaxing bioactive hydrogels
Source: Nat Commun. 2023 Oct 23;14:6604. doi: 10.1038/s41467-023-41907-1 (PMC10593748; doi:10.1038/s41467-023-41907-1)
Supplement: Supplementary file 2 — Reporting Summary [file 41467_2023_41907_MOESM2_ESM.pdf]

nature portfolio

Corresponding author(s): Kristopher Kilian
Last updated by author(s): Sep 21, 2023

Reporting Summary

Nature Portfolio wishes to improve the reproducibility of the work that we publish. This form provides structure for consistency and transparency in reporting. For further information on Nature Portfolio policies, see our Editorial Policies and the Editorial Policy Checklist.

Statistics

For all statistical analyses, confirm that the following items are present in the figure legend, table legend, main text, or Methods section.

n/a	Confirmed	
☐	☒	The exact sample size (*n*) for each experimental group/condition, given as a discrete number and unit of measurement
☐	☒	A statement on whether measurements were taken from distinct samples or whether the same sample was measured repeatedly
☐	☒	The statistical test(s) used AND whether they are one- or two-sided<br>*Only common tests should be described solely by name; describe more complex techniques in the Methods section.*
☒	☐	A description of all covariates tested
☒	☐	A description of any assumptions or corrections, such as tests of normality and adjustment for multiple comparisons
☐	☒	A full description of the statistical parameters including central tendency (e.g. means) or other basic estimates (e.g. regression coefficient) AND variation (e.g. standard deviation) or associated estimates of uncertainty (e.g. confidence intervals)
☐	☒	For null hypothesis testing, the test statistic (e.g. *F*, *t*, *r*) with confidence intervals, effect sizes, degrees of freedom and *P* value noted<br>*Give P values as exact values whenever suitable.*
☒	☐	For Bayesian analysis, information on the choice of priors and Markov chain Monte Carlo settings
☒	☐	For hierarchical and complex designs, identification of the appropriate level for tests and full reporting of outcomes
☒	☐	Estimates of effect sizes (e.g. Cohen's *d*, Pearson's *r*), indicating how they were calculated

Our web collection on statistics for biologists contains articles on many of the points above.

Software and code

Policy information about availability of computer code

Data collection	Avogadro 1.2.0 was used to create all .pdb peptide structure files. MARTINI 2.2 and GROMACS 2021.3 was used to prepare and run all MD simulations. Chirascan ProData Software was used to take and process all CD spectra. The Igor Pro software package and Quokka SANS reduction macros were used to process raw neutron scattering data. RheoCompass 1.30 (Anton Paar) was used to acquire rheological measurements from a MCR 102e rheometer. Zen Blue 2.5 (Zeiss) was used to acquire images from Zeiss LSM 800 confocal microscope. cellSENS Entry 1.15 (Olympus) was used to acquire images from Olympus DP73 optical microscope.
Data analysis	VMD 1.9.4 was used to visualize all MD simulations. Fiji-ImageJ (3) software was used to process images and extract quantitative data for analysis. SasView was used to fit models to the scattering data. The NIST Center for Neutron Research online scattering length density (SLD) calculator (May 2022 version) was used to calculate SLDs of peptides. Label-free protein quantification was performed using the MaxLFQ algorithm with default parameters. MaxQuant (2.1.3) was used to analyze raw peak lists of proteomics data, with the Andromeda algorithm. The human Swiss-Prot database (August 2022 release) was used to search peaks. Downstream analysis of quantified proteomics data was performed using the DEP R Package. Canonical pathway analysis of differentially abundant proteins was performed using QIAGEN IPA (September 2022 release). GraphPad Prism (9.5.0) software was used for statistical analysis and data visualization. ChemDraw (18.1) software was used for chemical drawing.

For manuscripts utilizing custom algorithms or software that are central to the research but not yet described in published literature, software must be made available to editors and reviewers. We strongly encourage code deposition in a community repository (e.g. GitHub). See the Nature Portfolio guidelines for submitting code & software for further information.

Data

Policy information about availability of data

All manuscripts must include a data availability statement. This statement should provide the following information, where applicable:

- Accession codes, unique identifiers, or web links for publicly available datasets
- A description of any restrictions on data availability
- For clinical datasets or third party data, please ensure that the statement adheres to our policy

The datasets generated and/or analyzed in this study are available in the ProteomeXChange Consortium under accession code PXD038870, and from the corresponding author upon request. The scripts used for MD simulations can be found at https://github.com/Ash-Nguyen/Trpzip.

Human research participants

Policy information about studies involving human research participants and Sex and Gender in Research.

Reporting on sex and gender	N/A
Population characteristics	N/A
Recruitment	N/A
Ethics oversight	N/A

Note that full information on the approval of the study protocol must also be provided in the manuscript.

Field-specific reporting

Please select the one below that is the best fit for your research. If you are not sure, read the appropriate sections before making your selection.

[x] Life sciences [ ] Behavioural & social sciences [ ] Ecological, evolutionary & environmental sciences

For a reference copy of the document with all sections, see nature.com/documents/nr-reporting-summary-flat.pdf

Life sciences study design

All studies must disclose on these points even when the disclosure is negative.

Sample size	No specific hypothesis or calculation of sample sizes was performed. Sample sizes were based on optimal density of cells or organoids for 3D culture with a minimum of n=3 technical replicates, across three different organoid lines with repeated independent experiments performed as necessary. Imaging of individual organoids were done with at least 20 images to represent the population of each condition.
Data exclusions	No data acquired for quantitative analysis were excluded.
Replication	All attempts at replication were successful. Live/dead viability was quantified across at least three independently prepared hydrogels. Bioprinted constructs were replicated at least three times and average diameter quantified across all printed constructs. Antimicrobial testing was performed on triplicate samples. Proteomics analysis of adult intestinal organoids was performed independently with three different patient-derived cell lines.
Randomization	All cell and organoid samples were randomly allocated to each experimental group.
Blinding	The same investigators both designed and performed experiments and data analysis, therefore there was no blinding concerning sample identity.

Reporting for specific materials, systems and methods

We require information from authors about some types of materials, experimental systems and methods used in many studies. Here, indicate whether each material, system or method listed is relevant to your study. If you are not sure if a list item applies to your research, read the appropriate section before selecting a response.

Materials & experimental systems

n/a	Involved in the study
☐	☒ Antibodies
☐	☒ Eukaryotic cell lines
☒	☐ Palaeontology and archaeology
☒	☐ Animals and other organisms
☒	☐ Clinical data
☒	☐ Dual use research of concern

Methods

n/a	Involved in the study
☒	☐ ChIP-seq
☒	☐ Flow cytometry
☒	☐ MRI-based neuroimaging

Antibodies

Antibodies used

Primary antibodies for immunofluorescence staining:
CDX2 – rabbit monoclonal antibody (1:300; Abcam; ab76541)
MUC2 – mouse monoclonal antibody (1:300; Invitrogen; MA5-12345)
Lysozyme – rabbit monoclonal antibody (1:300; Abcam; ab108508)
ZO-1 – rabbit polyclonal antibody (1:300; Abcam; ab216880)
Collagen I – rabbit polyclonal antibody (1:300; Abcam; ab34710)

Secondary antibodies for immunofluorescence stainings:
Anti-rabbit IgG (H+L), CF 647 antibody produced in goat (1:500; Sigma-Aldrich; SAB4600184).
Anti-mouse IgG (H+L), CF 555 antibody produced in goat (1:500; Sigma-Aldrich; SAB4600066).
Hoescht 33342 (1:500; ThermoFisher Scientific; H3570).
Phalloidin-Atto 488 (1:500; Sigma-Aldrich; 49409).

Validation

CDX2 – The manufacturer recommends using the antibody for WB and IHC-P applications, however at the time of purchase (2021) the antibody was recommended for ICC/IF applications. The antibody has been cited in 110 publications, some of which involve using the antibody for IF applications (https://www.abcam.com/products/primary-antibodies/cdx2-antibody-epr2764y-ab76541.html).
MUC2 – The manufacturer recommends using the antibody for ICC/IF and other applications. The antibody has been cited in 13 publications (https://www.thermofisher.com/antibody/product/MUC2-Antibody-clone-996-1-Monoclonal/MA5-12345).
Lysozyme – The manufacturer recommends using the antibody for ICC/IF and other applications. The antibody has been cited in 81 publications (https://www.abcam.com/products/primary-antibodies/lysozyme-antibody-epr29942-ab108508.html).
ZO-1 – The manufacturer recommends the antibody for WB and ICC applications, however at the time of purchase (2021) the antibody was recommended for ICC/IF applications. The antibody has been cited in 85 publications (https://www.abcam.com/products/primary-antibodies/zo1-tight-junction-protein-antibody-ab216880.html).
Collagen I – The manufacturer recommends using the antibody for WB applications only, however at the time of purchase (2019) the antibody was recommended for ICC/IF applications. The antibody has been cited in 1659 publications, some of which involve using the antibody for IF applications (https://www.abcam.com/products/primary-antibodies/collagen-i-antibody-ab34710.html).

Eukaryotic cell lines

Policy information about cell lines and Sex and Gender in Research

Cell line source(s)

HFF-1 cells (human fetal fibroblasts) were purchased from ATCC at passage 13 (SCRC-1041; lot number 60732085). C2C12 cells (mouse myoblasts) were purchased from ATCC (CRL-1772). Adult stem cell derived intestinal organoids were sourced from the molecular and integrative Cystic Fibrosis (miCF) Biobank (HREC16/SCH120) and were previously established from crypts isolated from four to six rectal patient biopsies. Human small intestinal organoids were derived from hepatic fibroblast derived human induced pluripotent stem cells (HYS0103, ACS-1020, lot number 0176; ATCC) using the STEMdiff small intestinal organoid kit (StemCell Technologies; cat #05140).

Authentication

HFF-1 cells, C2C12 cells, and human iPSCs had been authenticated through STR profiling by ATCC prior to purchase, as confirmed through the Certificate of Analysis provided. Morphology of all cell lines under brightfield imaging upon receipt of cells was additional authentication. HFF-1s were additionally authenticated via species-specific PCR primers. iPSCs were additionally authenticated by karyotyping by ATCC. Pluripotency of iPSCs was verified by immunofluorescence stainings of known pluripotency factors.

Mycoplasma contamination

All cell lines used in this study tested negative for mycoplasma contamination.

Commonly misidentified lines
(See ICLAC register)

No commonly misidentified cell lines were used in this study.
