# Supplementary material for: Hierarchical assembly of tryptophan zipper peptides into stress-relaxing bioactive hydrogels
Source: Nat Commun. 2023 Oct 23;14:6604. doi: 10.1038/s41467-023-41907-1 (PMC10593748; doi:10.1038/s41467-023-41907-1)
Supplement: Supplementary file 8 — Description of Additional Supplementary Files [file 41467_2023_41907_MOESM8_ESM.pdf]

Title: Supplementary Movie 1.
Description: Video recording of Trpzip gel prepared at 1% (w/v) in DMEM, pH 7 after gelation overnight at 37 ºC.

Title: Supplementary Movie 2.
Description: Brightfield live imaging over 90 minutes of organoid response to treatment with the CFTR modulator Trikafta in Matrigel.

Title: Supplementary Movie 3.
Description: Brightfield live imaging over 90 minutes of organoid response to treatment with the CFTR modulator Trikafta in pure Trpzip hydrogels.

Title: Supplementary Movie 4.
Description: Brightfield live imaging over 90 minutes of organoid response to treatment with the CFTR modulator Trikafta in Trpzip gels with low laminin content.

Title: Supplementary Movie 5.
Description: Brightfield live imaging over 90 minutes of organoid response to treatment with the CFTR modulator Trikafta in Trpzip gels with high laminin content
